# Mouse Models of Liver Parenchyma Injuries and Regeneration

**DOI:** 10.3389/fcell.2022.903740

**Published:** 2022-05-05

**Authors:** Yuan Du, Wencheng Zhang, Hua Qiu, Canjun Xiao, Jun Shi, Lola M. Reid, Zhiying He

**Affiliations:** ^1^ Department of General Surgery, Ji’an Hospital, Shanghai East Hospital, School of Medicine, Tongji University, Ji’an, China; ^2^ Institute for Regenerative Medicine, Shanghai East Hospital, School of Life Sciences and Technology, Tongji University, Shanghai, China; ^3^ The First Affiliated Hospital of Nanchang University, Nanchang, China; ^4^ Shanghai Institute of Stem Cell Research and Clinical Translation, Shanghai, China; ^5^ Shanghai Engineering Research Center of Stem Cells Translational Medicine, Shanghai, China; ^6^ Departments of Cell Biology and Physiology, Program in Molecular Biology and Biotechnology, UNC School of Medicine, Chapel Hill, NC, United States

**Keywords:** mouse models, liver diseases, liver regeneration, cell transplantation, cell therapies, transgenic mice

## Abstract

Mice have genetic and physiological similarities with humans and a well-characterized genetic background that is easy to manipulate. Murine models have become the most favored, robust mammalian systems for experimental analyses of biological processes and disease conditions due to their low cost, rapid reproduction, a wealth of mouse strains with defined genetic conditions (both native ones as well as ones established experimentally), and high reproducibility with respect to that which can be done in experimental studies. In this review, we focus on murine models for liver, an organ with renown regenerative capacity and the organ most central to systemic, complex metabolic and physiological functions for mammalian hosts. Establishment of murine models has been achieved for all aspects of studies of normal liver, liver diseases, liver injuries, and regenerative repair mechanisms. We summarize key information on current mouse systems that partially model facets of clinical scenarios, particularly those associated with drug-induced acute or chronic liver injuries, dietary related, non-alcoholic liver disease (NAFLD), hepatitis virus infectious chronic liver diseases, and autoimmune hepatitis (AIH). In addition, we also include mouse models that are suitable for studying liver cancers (e.g., hepatocellular carcinomas), the aging process (senescence, apoptosis), and various types of liver injuries and regenerative processes associated with them.

## Introduction

Animal models, especially mouse models, with the wealth of ones defined genetically and characterized extensively, are unique and irreplaceable in the field of regenerative medicine. *Ex vivo* models (2D, monolayer cell cultures or 3D ones such as spheroids, organoids, and bioartificial organs) are extremely important to complement those *in vivo* but, on their own, are unable to incorporate all the variables associated with disease pathogenesis, new drug screening and evaluations, the establishment of new treatment methods, and the evaluation of disease treatment efficacy. In summary, it is important to make use of both *ex vivo* models, facilitating focus on specific variables, and *in vivo* models enabling an overall perspective of factors that together influence a biological process and/or disease state.

Liver, as the primary organ of metabolism and systemic regulation, is critical for regeneration, for the intervention and repair, and inseparable from the establishment of evaluation systems based on liver injury models. In the past decades, the classical models of hepatectomy and toxin-induced liver damage have been used to simulate basic processes of liver regeneration (Mao et al., 2014; Forbes and Newsome, 2016). Recent studies have described signaling pathways occurring during liver injury and regeneration. However, complex processes involving paracrine signaling, the crosstalk between parenchymal and non-parenchymal cells, remain to be explored (Campana et al., 2021). Thanks to the development of lineage tracking technology, single cell sequencing and whole genome sequencing technology, new ideas have emerged that are promising avenues for resolution of questions and insights into these remaining areas of interest and associated problems.

Numerous rat and murine models have been used to study the mechanism of acute and chronic liver injury and are the most widely used (Forbes and Newsome, 2016). Although rodent models do not always perfectly mimic the clinical conditions relevant to liver and biliary diseases, their research value for studies in cell repair and organ regeneration is widely accepted for acute and chronic liver injuries. This review summarizes advantages and disadvantages of the commonly used mouse models of liver injury, while acknowledging research related to clinical translation of these models. This further gives recognition to the unsolved problems in these fields, indicating possible strategies for further optimization and establishment of new mouse models of liver injury and regeneration.

## Mouse Models of Acute Liver Injury

### Acute Liver Injury

Acute liver injury in humans is manifested early by massive necrosis of the liver parenchyma, decreased liver functions, and elevated levels of transaminases in the serum (Crismale and Friedman, 2020). The causes of injury are varied and include: 1) injuries selectively affecting hepatocytes, including damage by alcohol, acetaminophen (the most common drug-induced liver damage), antibiotics, viral hepatitis, and liver resection. The injuries in all of these are indicated clinically by the level of aspartate aminotransferase (Eslam et al., 2020) and alanine aminotransferase (ALT) in the blood; 2) bile duct-damaged liver injuries, including gallstones, biliary tract tumors, congenital biliary malformations, and parasites. The level of bilirubin in serum is often used as the clinical diagnostic index for these injuries (Mariotti et al., 2018).

The choice of an ideal animal model is influenced by the purpose of the study and, more importantly, on a clear clinical criterion (O'Grady et al., 1989). So far, surgical resection is still a state-of-the-art technique used as a classic process to model liver injury, with the hepatic resection model being the most common mouse animal model mimicking acute liver injury, while another, the ischemic ligation devascularization model, has also been used in some studies as a more conservative alternative (Table 1). According to the difference in liver volume resection, it can be roughly divided into 70% and 90% volume liver resection, which simulates the successful regeneration process after relatively limited liver damage and the decompensation period after fulminant liver failure, respectively.

**TABLE 1:** Mouse models of acute liver injury.

Clinical scenarios	Mouse model	Method procedures	Pathological changes	Types of injuries	Strengths	Weaknesses	References
Partial liver resection (various benign and malignant diseases that cause hepatic resection)	70% partial hepatectomy	Resect the left and middle lobes	Hemodynamic changes in the portal vein, vascular endothelial damage, involvement in hepatocyte hyperplasia, hypertrophy, inflammatory cell infiltration	Acute injury (compensatory); liver regeneration	Simple, reproducible, easy for evaluation and observation	Clinical scenario application limited	Mitchell and Willenbring, (2008)
Liver failure	70–90% hepatectomy	Resect the left, middle and partial right lobes	Massive hepatocytes necrosis; DAMPs-related immune reaction; decreased liver functions; abnormal coagulation functions	Acute liver injury (decompensated); liver failure	Easy for evaluation and observation	Irreversible injury,	Govil (2020), Bernal and McPhail (2021)
Small-for-size Syndrome (SFSS)					Excellent reproducibility including hepatic encephalopathy	Short survival cycle	
Drug-induced liver injuries (Acute)	Drug-induced liver injuries	Fasting for 12 h, then administration of 250–300 mg/kg APAP, intraperitoneal or caudal vein	Mitochondrial poisoning-induced hepatocyte damage; exacerbates by activating the immune responses	Acute liver injury; liver failure	Ideal reproducibility, good clinical consistency	Dose-dependent, APAP metabolism complexity	Mitchell et al. (1974)

Protocols of hepatectomy surgery were first established in 1931. Higgins and Anderson achieved a total liver volume equivalent to approximately 70% by resecting the left and middle lobes of the rat liver (Higgins and Anderson, 1931). This protocol of liver surgery has been widely used in mice, dogs, pigs and other mammals. The detailed descriptions of the original version of the surgical process are very limited. The primary purpose of the protocol has been to describe the liver regenerative process following removal of a portion of the liver and in which the remaining liver tissue contains all maturational lineage-dependent ploidy stages of parenchyma (from diploid to various polyploid stages); after the hepatectomy, the remaining diploid parenchymal cells undergo complete cell division, whereas the remaining polyploid ones undergo nuclear division, but not cytokinesis, followed by parenchymal cell hypertrophy. Therefore, although different laboratories have established their own hepatic resection protocols, the results from different laboratories often vary greatly (Wüstefeld et al., 2000; Greene and Puder, 2003; Borowiak et al., 2004; Martins et al., 2008). Until 2008, Claudia Mitchell et al. proposed an acute liver damage model construction protocol in which there is removal of 2/3s of the liver by a rapid surgical procedure requiring 15–20 min (Mitchell and Willenbring, 2008). The repeatability and host tolerance of the protocol have been widely recognized in the field. On this basis, Nevzorova, Y. A et al. made further protocol improvements and proposed a standardized protocol for partial liver resection in mice in 2015 (Nevzorova et al., 2015).

Classic experiments in mice have shown the accessibility and ease of experimental manipulation of partial hepatectomy as an ideal way to study the mechanism of liver regeneration (Michalopoulos and Bhushan, 2021). After liver resection, the remaining liver enters a pre-proliferative state from a resting state, followed by active proliferation of hepatocytes from the G1 stage (0–6 h post-hepatectomy) to the S stage (6–24 h, DNA replication) and then to the M stage (cell mitosis) (Wang et al., 2003; Klochendler et al., 2012). This procedure triggers DNA synthesis in all the parenchymal cells accompanied by complete cell division in the periportal, diploid cells, whereas in the polyploid cells, there is further increase in polyploidy, an absence of cytokinesis and hypertrophy. The hypertrophic cells undergo more rapid apoptosis and senescence, are cleared and replaced with cells derived from the periportal diploid cells. This process requires, on average about 4–5 weeks (depending on the species).

These findings are distinct from those triggered by selective loss of parenchymal cells at a particular ploidy stage. Selective loss of the diploid subpopulations results in fibrotic responses and in cirrhosis, whereas selective loss of the polyploid cells results in rapid hyperplasia with complete cell divisions in the remaining diploid parenchymal cells, as occurs with the effects of pericentral injuries (e.g., carbon tetrachloride or CCl4, radiation, or certain viruses) (Sigal et al., 1999).

Both models are representative of findings in patients. The partial hepatectomy model simulates natural progression after hepatectomy surgery in patients with various liver diseases and explores novel therapeutic measures and enhance the recovery after hepatectomy, but also provide a more effective and safe guidance program for living donor liver transplantation (Nojima et al., 2016; Liang et al., 2021). The CCl4 model simulates conditions for patients subjected to toxins, to radiation or having viral infections that target the pericentral (polyploid) cells.

Further investigations of underlying regulation mechanisms of liver regeneration post partial hepatectomy highlight Hippo pathways. Shortly after partial hepatectomy, TNF and IL-6 secreted by non-parenchymal cells can activate hepatocytes by stimulating the intracellular NF-kB and STAT3, which activate the residual hepatocytes (Taub, 2004; Fausto et al., 2006). The expression level of TGFβ in those hepatocytes then increases followed by accumulations of pSmad2 and Yap1 in nuclei which enable the proliferation of hepatocytes in liver regeneration (Oh et al., 2018). By the end of liver regeneration, core kinases of the Hippo pathway, mammalian Sterile20-like (MST) 1 and 2, form feedback loop signaling, by controlling downstream effectors, Yes-associated protein (YAP), in order to regulate the size of the liver (Moya and Halder, 2019). Remarkably, these termination events also play a vital role, together with the Hippo pathway, in the maintenance of the standard liver mass, as well as cancer suppression post-PHx (Michalopoulos, 2017). Despite the fact that simultaneous genetic depletion of MST 1/2 yields embryonic lethality, MST1^−/−^ or MST2^−/−^ mice develop larger organ sizes and even form hepatocellular carcinomas. Another concern is HDAC, which has been proved to treat rare cancer and cell development. Previous studies have proved that HADC10 mediates the effect of malnutrition on liver weight (Pinto et al., 2016). Despite its close link with cancer, auxiliary functions in the regulation of organ size need further elucidation.

It is worth mentioning that the various signaling pathways involved in the regeneration of liver damage also play an important role in liver cancer. Consequently, some partial hepatectomy models can also be used as a tool to study the oncogenic process of liver cancer (Oh et al., 2018). However, we must also be aware that because the partial hepatectomy model itself simulates only a small number of clinically acute liver damage scenarios, those affecting all of the parenchymal cells, both diploid and polyploid ones.

In summary, liver responses to partial hepatectomy versus to pericentral toxins yield distinct responses that occur in liver regeneration, and both are important to an understanding of control of parenchymal cell proliferation. The former occurs with surgical resection of the liver but with the maturational lineage stages and their feedback loops remaining intact; the second one occurs with selective loss of the late maturational lineage stage (polyploid) cells and loss of the feedback loops that they generate.

### Acute Liver Failure

Despite the liver’s remarkable regenerative ability, when the damage of parenchymal cells exceeds the threshold of the liver’s capacity for regeneration, liver damage progressively transforms into acute liver failure and can be clinically accompanied by multi-organ failure, coagulation dysfunction, and hepatic encephalopathy; the mortality rate of patients in these circumstances increases sharply (Wendon et al., 2017; Bernal and McPhail, 2021). To date, the treatment of acute liver failure remains a major clinical problem, and the only effective strategy is orthotopic liver transplantation. Therefore, it is urgent to establish a class of efficient and reproducible models of acute liver failure for the establishment of therapeutic strategies. So far, the hepatectomy model (70–90%) versus the pericentral, hepatotoxic drug (paracetamol, CCl4)-induced model are still the mainstream mouse models of acute liver failure, while the construction of virus-induced liver failure models has not yet succeeded (Ding et al., 2010; Fujiwara and Nakamura, 2020; Kolodziejczyk et al., 2020). This is likely due to the fact that different viruses have distinct targets. For example, hepatitis A replicates in all ploidy stages of parenchymal cells versus hepatic B and C that replicate in diploid parenchymal cells and result in apoptosis and cell death in polyploid parenchymal cells (Bissig et al., 2010; Washburn et al., 2011; Yamane et al., 2014; Yamane et al., 2019).

In the past 20 years, the field of liver surgery has made great progress, and a variety of different liver resection procedures have been creatively developed and established, such as Associating Liver Partition and Portal Vein Ligation for Staged Hepatectomy (ALLPS), which provides novel strategies for patients with liver tumors (Schnitzbauer et al., 2012). However, the ensuing class of dangerous complications, small-for-size Syndrome (SFSS), cannot be ignored (Eshkenazy et al., 2014). When some patients have impaired their liver regenerative capacity (for example, if they have a history of cirrhosis) or requiring extensive liver resection, these patients are susceptible to postoperative residual liver insufficiency. In mouse models, when the volume of liver resection is increased to 90%, the hepatectomy model can mimic the SFSS-related symptoms and is accompanied by a sharp increase in mortality after surgery (Lehmann et al., 2012; Czigány et al., 2015; Forbes and Newsome, 2016; Govil, 2020). Consistent with clinical scenarios, due to individual differences, when the volume of the liver is removed beyond a certain threshold, the remaining liver regeneration function declines, eventually leading to the inevitable occurrence of liver failure (Ikegami et al., 2020a). Therefore, the study of 90% hepatectomy mouse models provides an ideal and important means for the further clarification of the pathogenesis of SFSS, as well as screening of different liver support systems for SFSS (Dili et al., 2019; Ikegami et al., 2020b) (Table 1).

In addition, a variety of hepatotoxic drugs can also induce acute liver failure. For example, acetaminophen (paracetamol)-induced liver damage is the most common cause of liver failure in clinic practice (Stravitz and Lee, 2019). Rodent modeling can also be performed using acetaminophen (Mao et al., 2014). Since acetaminophen is a class of dose-dependent drugs, it is converted by CYP2E1 into *N*- acetyl-*p*- benzoquinone imine (NAPQI), which progressively depletes the pool of glutathione, causing redox imbalance (Mitchell et al., 1974). However, due to the lack of standardized dosage and mode of administration, the degree of drug-induced acute liver injury is not easy to control precisely, which will not only cause different degrees of acute liver injury, but even lead to the occurrence of chronic liver injury (Woolbright and Jaeschke, 2017; Wang et al., 2021).

During the process of drug-induced acute liver injury, a number of reasons can lead to modeling failure include: age and sex of the animal, the use of a CYP450 inducer, etc. (Du et al., 2014; Maes et al., 2016). In addition, due to the large difference in the concentration of specific coagulation factors in the blood of rodents and humans, the pathological processes that may lead to acute liver failure in mice is significantly different from the clinical pathological characteristics of human patients (Groeneveld et al., 2020; Driever et al., 2021). These differences are very common in carbon tetrachloride (CCl4) modeling, and we will discuss this in more detail below with respect to CCl4 modeling chronic liver damage.

## Mouse Models of Chronic Liver Injury

Long-term chronic hepatotoxic substances, such as alcohol and metabolic diseases, are often the cause of chronic liver damage to the parenchymal cells of the liver. Congenital and acquired bile duct obstruction is also a mechanism of clinical chronic liver injury.

### Drug-Induced Liver Injury

In the clinical scenario, adverse drug reactions are closely related to patient morbidity, mortality, medical costs, and drug discovery failure rates. As an important metabolic and detoxification organ, the liver is also highly susceptible to hepatotoxic drugs (Norman, 2020). Drug-induced liver injury is a patient-specific, temporary, multifactorial, pathophysiological process. Although DILI causes about 50% of acute liver failure cases in the clinic, it also plays an important role in chronic liver injury, leading to related diseases such as autoimmune DILI, granulomatous hepatitis, and hepatic sinus obstruction syndrome (Dakhoul et al., 2018). The common clinical DILI can be attributed to damage to mitochondrial and lysosomal functions, obstruction of bile excretion, endoplasmic reticulum stress, and disorders of the innate immune and adaptive immune systems. So, it is a complex process with multi-stage, multicellular participation, which also poses a challenge to the establishment of animal models.

**FIGURE 1:**
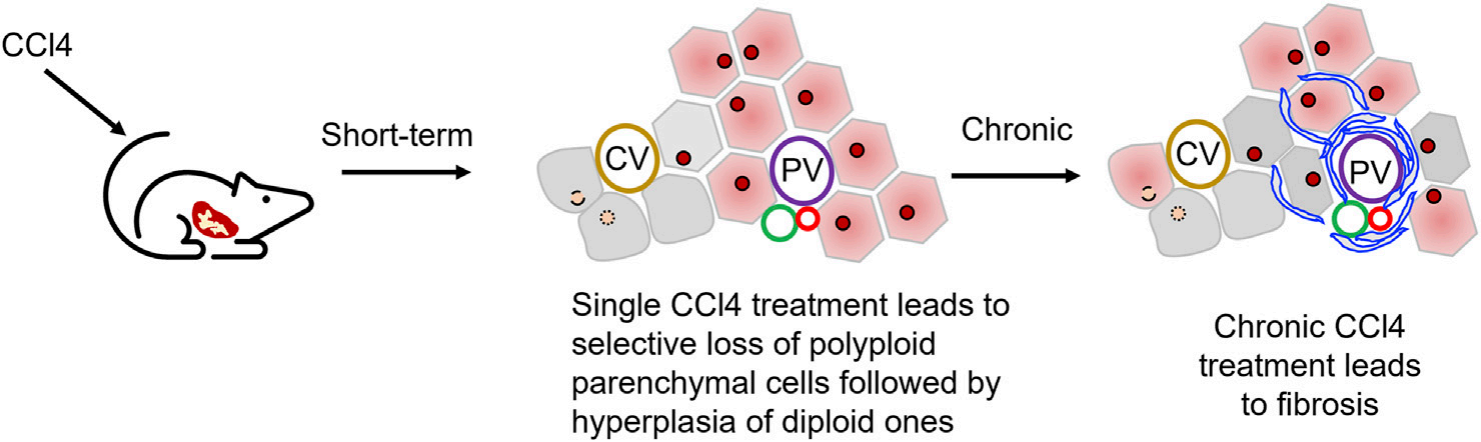
Use of carbon tetrachloride, CCl4, to establish liver injury models. CCl4 administration leads to the damages of hepatocytes in hepatic central zone. Normally a single dose of CCl4 will show acute liver injury phenotype as pericentral necrosis and steatosis, while the prolonged administration causes fibrosis, cirrhosis, or HCC.

The metabolic damage pathway for CCl4 is relatively clear, and it is currently the most used induction drug for the establishment of animal models of liver fibrosis and cirrhosis (Table 2). As noted above, CCl4 targets pericentral parenchymal cells, those that are polyploid; the loss of these pericentral cells, followed by loss of the feedback loop signals, triggers a hyperplastic response, one of complete cell division, by the diploid, periportal parenchymal cells (Figure 1). Indeed, although the CCl4 modeling mechanism is quite different from the complex mechanism of liver damage in clinics, this model can mimic the characteristics of clinical DILI-related chronic liver disease to a certain extent, which has extensive research value. CCl4 can produce trichloromethyl radicals through the liver’s CYP2E1 biotransformation pathway, accompanied by oxygen radicals and lipid peroxidation processes, resulting in damage to the central vein-dominated, polyploid parenchymal cells combined with local inflammation (Li et al., 2015; McGill and Jaeschke, 2019). Its hepatotoxicity generally peaks at 24 h after administration. Repeated use of CCl4 activates Kupffer cells, activates hepatic stellate cells into scarring myoblasts, leading to the onset and progression of hepatic fibrosis (Issa et al., 2003). With the deposition of excess liver scar tissue, it eventually develops into nodular cirrhosis (Iredale, 2007). When CCl4 is discontinued, the liver parenchyma regenerates, accompanied by partial degradation of scars and regression of inflammation. Therefore, the model is reversible.

**TABLE 2:** Administration of carbon tetrachloride (CCl4) for the liver injury mouse model.

Clinical scenarios	Method procedures	Pathological changes	Phenotypes/Outcomes	Strengths	Weaknesses	References
Drug- induced liver injuries (Acute)	100 mg/kg, intraperitoneal injection, single dose	Acute hepatotoxic injury caused by oxidative stress	Acute liver injury; liver failure	Good repeatability, easy modeling	Dose dependence	Li et al. (2015)
Drug-induced liver injuries (Chronic)	100–150 mg/kg, Intraperitoneal injection, 2 to 3 times a week for 4–6 weeks	Central vein-dominated injury; activation of stellate cells to myofibroblasts	Chronic liver injury; fibrosis, carcinoma	Short modeling time; present significant hepatic steatosis	Lacking standard operation, reversible fibrosis	Forbes and Newsome, (2016)

During model development, the CCl4 effect has a strain-dependent feature, and BALB/C has more advantages than C57BL/6 and DBA/2 in CCl4-modeled liver fibrosis (Shi et al., 1997). However, due to the dose dependence and the variety of routes of administration, the standard CCl4 modeling even within the same strain is also controversial. At present, the most commonly used experimental protocol is intraperitoneal injection 2 to 3 times a week for 4–6 weeks, and the dose of administration is 500–700 μl/kg (McGill and Jaeschke, 2019). Similarly, CCl4 can also be administered by oral, subcutaneous, inhalation and other routes, each of which has its own advantages and disadvantages. Scholten et al. (2015) believe that oral administration increases the mortality rate of animal molding. As an emerging route of administration in recent years, inhalation has the characteristics of short dosing time and stable drug concentration, but its own high requirements for specialized equipment limit widespread use and popularization (Nagano et al., 2007).

To date, animal models based on immune imbalance and gut microbiome-related LPS demonstrate different mechanistic steps leading to Idiosyncratic drug-induced liver injury. Revolutionized advancements provide insights to cancer therapy, however, they also bring immune related adverse reactions in multiple organs such as liver. Two major immune-check point inhibitors, including programmed cell death protein-1/ligand-1 (PD-1/PD-L1) and cytotoxic T-lymphocyte-associated antigen 4 (CTLA-4) are dominant contributors in balancing immune response and tolerance. An important side effect of ICIs is significantly increase in immune response, thereby breaking intrinsic immune tolerance in liver. Administration to PD-1^−/−^ mice of anti-CTLA-4 leads to tardive onset of liver injury with parallel pathological changes from that of DILI in humans (Metushi et al., 2015). The other is less described LPS with respect to intestinal dysbiosis hypothesis, which is associated with exposure to LPS in human idiosyncratic DILI. However, the latter is widely divergent from the clinical condition (Roth et al., 1997; McGill and Jaeschke, 2019). In essence, the unpredictable nature of Idiosyncratic DILI needs further exploration; new ideas like reactive metabolites and exosomes are worthy of attention (Teschke and Uetrecht, 2021).

Overall, despite the various advantages and disadvantages of the CCl4 model, it is still a widely used chronic liver injury model. With chronic administration of CCl4, different cell types involved in the process of liver fibrosis and the activated signaling pathways can be studied. Also, when the drug is withdrawn, the process of liver fibrosis and inflammation resolution can be observed, especially the key role played by cell types with strong plasticity such as macrophages. However, due to dose-dependent effects and reversible damage, it is difficult to apply it to the evaluation of cell hepatic regeneration ability and drug mechanisms of action.

### Non-Alcoholic Liver Disease

In recent years, the global incidence of NAFLD and the rapid increase in related mortality have aroused widespread concern. NAFLD is a broad spectrum of diseases that encompasses simple fatty liver and nonalcoholic hepatitis (NASH), which can progress to cirrhosis and liver cancer. In Western countries, NAFLD patients are mostly associated with metabolic diseases such as insulin resistance (IR), obesity, and type 2 diabetes. Thus, in recent years, some scholars have proposed to change the name to MAFLD (metabolically associated fatty liver disease) (Eslam et al., 2020). The lack of effective therapeutic drugs and public health prevention strategies makes it difficult to prevent and treat NAFLD. At the same time, the lack of relevant animal models of diseases has also seriously hindered basic translational research related to NAFLD.

As a class of animal models applied to clinical translational research, it should be as far as possible in line with various pathological changes and external pathogenic risk factors in the natural occurrence of human NAFLD disease. From this point of view, diet-induced obesity is the best option (Table 3). In the process of modeling diet influences, the required dietary intake required by animal induction should be simulated as much as possible to that in the human diet with avoidance of alcohol or liver toxins, etc. that can contribute by distinct mechanisms. So it is better to simulate human NAFLD characteristics with conditions for obesity, insulin resistance, and systemic inflammation (Jahn et al., 2019). The liver phenotype of the NAFLD model should contain hepatic steatosis, lobular degeneration, hepatocyte swelling, and ideally the formation of Mallory bodies and hepatic fibrosis (Santhekadur et al., 2018). The MCD diet and the CDAA (Choline-Deficient L-Amino-Defined) diet are the two classic diet models, but both have poor metabolic parameter simulations (Kodama et al., 2009; Pierce et al., 2015).

**TABLE 3:** Mouse models of non-alcoholic fatty liver diseases (NAFLD).

Mouse model	Method procedures	Pathological changes	Types of injuries	Strengths	Weaknesses	References
Methionine choline-deficient (MCD) diet, choline-deficient, L-amino acid-defined (CDAA) diet	High-fat choline deficiency diet, fat content increased from 10% to 60% for 2–4 weeks	Change of carbohydrate metabolism, no insulin resistance; increased fatty acid intake and fibrosis	Chronic liver injury; fibrosis	Short modeling time, significant hepatic steatosis	Lacking insulin resistant, distinguished parameters with human NAFLD.	Pierce et al. (2015)
		Chronic liver injury; fibrosis				
*ob/ob* or *db/db* mouse	Leptin (ob/ob)or leptin receptor knock out(db/db)	Organ fat redistribution, insulin resistance, obesity, hepatocyte lipotoxicity and apoptosis;	Chronic liver injury, without fibrosis (*ob*), fibrosis	Good reproducibility, obesity and insulin resistance	No fibrosis or partial fibrosis, requires in corporation of specific diet	(Wang et al., 2020b; Suriano et al., 2021, Schnitzbauer et al., 2012)
		Chronic liver injury, without fibrosis (*ob*), fibrosis				
Alms-ko mouse	Alms knock out	Impaired intracellular transport and appetite regulation; obesity; insulin resistance	Chronic liver injury, fibrosis	Good reproducibility, obesity, insulin resistance, significant fibrosis in a high-fat diet.	Modeling influenced by mouse strains	Arsov et al. (2006)
		Chronic liver injury, fibrosis				

Despite NAFLD becoming the most rapidly growing indication for liver transplantation and a causal variable in the development of hepatocellular carcinomas, potential mechanisms involved in the transformation of metabolic-related disease to NAFLD remain elusive (Imajo et al., 2012). Leptin, a peptide hormone secreted primarily by adipose cells in white adipose tissue, plays an important role in regulating energy balance. The Leptin deficiency (*ob/ob*) mice has been established to provide new ideas for NAFLD modeling (Suriano et al., 2021). *Ob/ob* mice develop severe insulin resistance, characterized by the redistribution of fat from adipose cells to the liver and non-adipose tissue. However, the leptin gene mutations alone cannot induce NAFLD, special diets, such as MCD, are further needed, Nevertheless, *ob/ob* mice with MCD fail to induce the liver fibrosis phenotype (Javor et al., 2005). In addition, relevant clinical studies have proved that leptin levels in human NAFLD are mostly normal or slightly elevated (Safar Zadeh et al., 2013; Polyzos et al., 2015), which also limit the application of leptin deficiency in mice.

A similar mouse model has been described, named leptin receptor deficiency (*db/db*) mice, in the development of NAFLD animal models (Suriano et al., 2021). Through interference with the leptin pathway, these mice develop insulin resistance and dyslipidemia. Likewise, both require special diets to induce NAFLD. However, the latter compensates for the deficiencies in liver fibrosis (Wang Q. et al., 2020). Indeed, the underlying differences between leptin and leptin receptor deficiency are not yet explored, as details associating leptin signals and metabolic disorder remain poorly understood, especially those related to liver fibrosis or cancers.

The ALMS1 gene that encodes for a ubiquitously expressed protein has been proved to be associated with cell cycling and energy metabolism. Mutations in the Alms1 gene can cause Alstrom syndrome in humans. When mice spontaneously lose 11 base pairs (foz/foz) from the Alms1 gene, combined with a high-fat diet, the mice show features of excessive obesity, insulin resistance, hepatomegaly, diabetes mellitus, high serum alanine transaminases levels, accompanied with bulk hepatocyte swelling, and peri-cell and pericentral fibrosis (Arsov et al., 2006; Heydet et al., 2013). Conversely, when one eliminates the high-fat diet, the inflammation of the liver does not completely subside. It can be used to simulate the transition from clinical NAFLD to NASH, which has a significant advantage, similar to that of NASH in the clinic. However, each characteristic that proved irreversible and its time-consuming features reduced its credibility in the basic research.

### Liver Disease Associated with Viral Infection

Common hepatitis virus families include A, B, C, D, E, and F, of which B (HBV), C (HCV), and E (HEV) have been characterized by the most in-depth studies. Despite the universal availability of the HBV vaccine, people living with hepatitis B virus still account for 1/3 of the world’s population, and about 240 million people are suffering from chronic infection with hepatitis B virus (Shi and Zheng, 2020). Current antiviral therapies have had little effect on chronic hepatitis B infection, due to the host specificity of human hepatitis B virus (Hu et al., 2019). However, natural infections have been rarely reported in rodent animals, which hampered the development of animal models related to hepatitis B virus. Thanks to the vigorous development of transgenic mouse technology, new solutions to the above problems have been provided.

Features of chronic HBV infection in humans include the formation of cccDNA (covalently closed circular DNA), the assembly and transmission of infectious viral particles, and persistence (Shih et al., 2018). In contrast to other mammals, humans have developed a unique immune system, due to the lack of an innate immune response to HBV (including interferons, interleukins, tumor necrosis factor, etc.) (Wieland et al., 2004). Unlike the dietary and drug liver damage models mentioned above, the progression of disease after chronic hepatitis virus infection is closely related to immunity. Immune (innate and adaptive immune) responses include antigen presentation, seroconversion of HBeAg (e antigen) and HBsAg (surface antigen), and the final result is virus clearance and virus tolerance, which leads to chronic liver damage and gradually progresses to cirrhosis and liver cancer.

Transgenic mice infected with hepatitis B virus do not initiate an innate immune response to clear the hepatitis B virus (Chisari et al., 1986) (Table 4). The hepatitis B virus particles produced by mouse hepatocytes are morphologically similar to those of human origin viral particles and can still mimic the pathological process of human infection with hepatitis B virus (Guidotti et al., 1995). However, liver fibrosis and cancer have never been observed in transgenic mice, and HBeAg or HBsAg have not been detected in murine serum (Kim et al., 1991). Transfected mice are constructed to avoid the above defects in the model. Through repeated hydrodynamic injection in the tail vein, the viral vector or adenovirus-containing HBV DNA is injected into the body, which results in a blunted rise of viral load in the serum. Subsequently, adaptive immune systems are activated to elicit virus clearance, which can achieve persistent hepatitis B virus infection (Yao et al., 2020).

**TABLE 4:** Mouse models of infectious liver damage.

Mouse model	Method procedures	Pathological changes	Types of injuries	Strengths	Weaknesses	References
HBV transgenic mice	Transgenic mice infected with DNA of HBV	Producing HBV-associated protein	Chronic liver injury; fibrosis	Good consistency with human hepatitis B	Without fibrosis, only a few strains of mice express hepatitis B surface antigen	Chisari et al. (1986)
Transfected mice	Continuous tail vein high pressure injection of HBV DNA	Hepatitis B infection and liver fibrosis	Chronic liver injury; fibrosis	High viral DNA concentration, sustainable injection	HbeAg seroconversion may be present, but no liver disease is present	Yao et al. (2020)
Humanized *Fah* ^*−/−*^ *Rag2* ^*−/−*^ *IL2rg* ^*−/−*^ mouse	Stepwise transplant in immunodeficient FRG	Recolonization of human hepatocytes in parallel with HBV-associated proteins	Chronic liver injury, fibrosis	Highly clinical consistency, mimicking the human immune system, controllable liver injury	Low viral DNA replication and human hepatocyte count	Sun and Li, (2017)

Another approach is use of humanized mice based on the successful engraftment of human hepatocytes that can truly simulate the pathological changes of human hepatocytes after infections. The most common humanized mice include: SCID (severe combined immunodeficiency) mice and *FRG* (*FAH*, fumaryl acetoacetate hydrolase, and *RAG2* dual knockout) mice.

SCID mice express urokinase-type plasminogen activators, resulting in subacute liver failure in newborn mice with renal and hematologic diseases. Meanwhile, this also provides the necessary environment in the host for the transplantation of human hepatocytes. However, due to the low fertility of the mice and the transient operation window limit the usefulness of this model and application scenarios.

*FRG* triple-gene knockout mice have deficiencies in three genes: *Fah* ^−/−^ (involved in tyrosine metabolism); *Rag2* ^−/−^ (recombinant activation gene 2-restrictive expression in developing lymphocytes, an important part of adaptive immunity); and *Il2rg* ^−/−^ (interleukin receptor γ chain). In the absence of *Fah*, the upstream gene generates hepatotoxic products causing hepatocyte death and kidney necrosis, and the other two genes provide an immunodeficient background that provides the basis for acceptance of xenotransplants of human hepatocytes. The drug, NTBC [(2-(2-nitro-4-trifluoromethylbenzoyl)-1,3-cyclohexanedione)] is used to bypass the genetic deficit in *Fah* hosts and so control the toxicity in them enabling survival of the hosts until it is desired to have effects that are evidence of the deficit of the FAH gene, produced by the simple withdrawal of the drug. Thus, it enables survival of the mice and expands their operating window of usefulness in the experimental studies.

Chimeric mice provide new ideas for the study of HBV cccDNA formation mechanisms and antiviral drug screening studies. The same system also provides important strategies for the study of HCV and HEV. In general, the humanized mouse model provides a completely new approach for the study of chronic hepatitis virus infection (i.e., HBV). However, due to the differences in the microenvironment of mouse and human parenchymal cells, distinctions in the proportion of parenchymal cells that are diploid versus polyploid (correlating with the number of engraftable parenchymal cells), and the low level of viral replication, further optimization is needed. It is also important to note that since *FRG* requires regular treatment of the mice with NTBC, this also limits the use of certain antiviral therapy drugs. Further advantages of studying hepatitis virus infection in liver-damaged mice can be extended when transplanting human hematopoietic stem cells and hepatic stem/progenitor cells, which provides a human immune system in mouse models for human parenchymal cells. This immune adaptive system simulates the pathological process after human liver infection with hepatitis virus in a more vivid way.

The role of hepatic viruses and maturational lineage mechanisms is worth mentioning. The liver in all mammals is in a maturational lineage extending from early stages located in the liver acinus at the portal triads (dominated by diploid cells) to end stages in cells near the central vein (and dominated by polyploid cells and apoptotic cells) (Sigal et al., 1992; Sigal et al., 1999). The final stage, Axin2^+^ diploid hepatocytes, linked on their lateral borders to the endothelia of the central vein, play roles in replacing the apoptotic cells at the end of the terminal differentiation process (Turner et al., 2011; Wang et al., 2015; Sun et al., 2020).

That lineage process has been extensively characterized by numerous investigators with characterization of the cells in the periportal (zone 1) versus midacinar region (zone 2) versus pericentral (zone 3) zones and the final stage, the Axin2^+^ diploid cells, mediating clearance of apoptotic cells. Some viruses (e.g., HBA) infect all maturational lineage stages equally; others (e.g., HBV and HCV) infect and proliferate best in early lineage stages (e.g., hepatoblasts and committed progenitors) but result in cell death in pericentral, polyploid parenchymal cells (Rehermann, 2013; Yamane et al., 2019). Thus, there are viruses (and also toxins) that can have effects that are maturationally lineage dependent (Tables 4, 6).

### Autoimmune Liver Disease

Autoimmune hepatitis (AIH) is a serious chronic liver disease with an increasing incidence worldwide in recent years. It is a progressive inflammatory liver disease characterized by chronic inflammation of the liver, circulating autoantibodies, hypergammaglobulinemia, and specific liver biopsy histologic features (interface hepatitis, rosettes, and lymphocyte invasion). Currently, immunosuppressive therapy is the standard clinical treatment for AIH, but side effects and recurrence in patients limit its application (Manns et al., 2010; Schmeltzer and Russo, 2018). Due to the lack of suitable mouse models, research on the pathogenesis of AIH is still limited.

In 1992, Tiegs et al. pioneered the use of concanavalin A (ConA) to build a mouse model of T-cell-mediated hepatitis, the most widely used tool to study immune-mediated liver injury (Tiegs et al., 1992). Subsequently, in ConA-treated mice, IFN-g (interferon-g) and TNF-a (tumor necrosis factor-a) were shown to be key mediators of liver damage (Kusters et al., 1996), similar to the situation in patients with AIH. However, the hepatitis in this mouse model is acute onset and usually disappears within 48 h. Features of AIH, such as the presence of autoantibodies, typical interfacial hepatitis and progressive hepatic fibrosis, are not observed in this model (Table 5).

**TABLE 5:** Mouse models of autoimmune hepatitis (AIH).

Mouse model	Method procedures	Pathological changes	Types of injuries	Strengths	Weaknesses	References
ConA	1.5 mg/kg ConA, intravenous injection	Activated T lymphocytes induced progressive hepatitis, lymphocyte infiltration; rapid increase of transaminase;	Acute or chronic liver injury	Good repeatability; produce immune mediators (IFN- γ etc.); easy to operate	Rapid, not in line with clinical chronic injuries	Tiegs et al. (1992)
		acute liver injury				
DNA immunization	50ul DNA vaccines of FTCD and CYP2D6,muscle injection	Cytotoxic T cells mediated hepatocyte necrosis; serum aminotransfer reaches a peak from 4 to 7 months;	Acute liver injury; liver failure	Close to clinic scenarios	Modeling results affected by mouse strain, gender and age	Lapierre et al. (2004)
HLA-DR3, DR4 transgenic mice	DNA plasmid induced antinuclear antibodies	Immune cell infiltration; liver fibrosis	Acute and chronic liver injury (depending on time and concentration of NTBC)	Good clinical consistency; autoantibody on specific T cells involved	Only transgenic male mice developed AIH, in contrast to females in human AIH.	Zierden et al. (2010)
Alb-HA/CL4-TCR transgenic mice	CL4-TCR transgenic CD8 (+) T cells were also adoptively transferred into Alb-HA mice	Spontaneous chronic autoimmune mediated hepatitis, necrotizing inflammatory lesions, liver fibrosis and elevated transaminase levels		Good repeatability; involves the study of HLA and AIH	Autoimmune susceptibility is restricted by strain	Yuksel et al. (2015)

Autoantibodies against hepatocytes play an important role in the pathogenesis of AIH. Therefore, taking known autoantigens into consideration to break down immune tolerance may provide a pathway for establishing a mouse model of chronic AIH. In type 2 AIH, CYP2D6 is one of the most characteristic autoantigens recognized by type 1 liver/kidney microsomal autoantibodies (LKM-1). In 2004, Lapierre et al. (2004) established a mouse model of AIH for the first time by DNA immunization of CYP2D6 and FTCD. Cytotoxic specific T cells and necrotizing inflammation were found, and alanine aminotransferase (ALT) levels peaked 4–7 months after injection. Anti-lkm1 and anti-LC1 antibodies were also detected in mouse serum, which were persistently elevated for at least 8 months. In 2013, Hardtke-Wolenski et al. (2017) established a type 2 AIH model by inducing self-limiting adenovirus infection by FTCD. The authors also demonstrated that the development of AIH in autoantibody-positive animals is determined by the genetic background. In addition, an improved method of CYP2D6-induced AIH mouse model was established by initial disposable adenovirus infection and repeated injection of human CYP2D6 plasmids using hydrodynamic liver-targeted gene delivery technology (Wang H. et al., 2020). Novel chimeric liver models AIH through DNA immunization of human HLA-DR3 transgenic mice and inducing chronic liver injury that closely mirrors human AIH (Yuksel et al., 2015). Similarly, adoptive transfer of CL4-TCR transgenic mice T cells into Alb-HA mice was performed to mimic human AIH (Westendorf et al., 2006; Zierden et al., 2010). The initial transient hepatitis is achieved by using multiple consecutive expression of pure adenovirus and naked CYP2d6 plasmids. Autoantibodies and interface hepatitis can be observed 4 weeks after the first injection, and progressive liver fibrosis occurs at 5 weeks. This provides a new technical approach for establishing a mouse model of CYP2d6-induced Type 2 AIH.

Treg is an important regulatory cell for maintaining immune tolerance and shows considerable potential in treating a variety of autoimmune diseases (Esensten et al., 2018). Depletion of Tregs in mice has been reported to build AIH mouse models. Animal studies have shown that improving the number of Tregs in the liver, or impaired Treg/Th17 balance, can reduce immune-mediated liver damage in mice (Hegde et al., 2008; Hu et al., 2018). In 2015, Hardtke-Wolenski et al. (2015) reported intrahepatic, high-proliferative Treg in a mouse model of spontaneous transgenic AIH with persistently severe AIH, and they also found that the AIH of these mice could be treated with Treg adoptive transfer. Although the mechanisms by which different mouse models trigger AIH differ, and the findings of different studies vary, Treg-related studies in a variety of different mouse models still can simultaneously help explain the process of autoimmune hepatitis.

Therefore, the use of different ways to induce mouse AIH can effectively simulate the clinical symptoms and serological manifestations of different degrees and different types of human AIH, and in addition, it also provides new targets and treatment ideas for the treatment of AIH.

## Mouse Models for Studying Liver Cell Transplantation in Treatment of Liver Injury Repair

Since various hepatoxic factors can induce acute or chronic liver injury, liver disease accounts for nearly 3.5% of all worldwide deaths (Asrani et al., 2019; Hu et al., 2020). Although clear evidence of therapeutic benefits of liver transplantation has been proved, it is still limited by the scarcity of organs, especially ones of reasonable quality, by expensive surgery and by the possibility of rejection by the host. Goals for future treatments of patients include cell therapies involving transplantation of isolated mature parenchymal cells or of stem/progenitors. Such therapies have long been desired but minimally explored because the many efforts to establish hepatic cell therapies using direct injection into the liver or injection *via* a vascular route, such as the portal vein into the liver, resulted in low efficiencies, typically under 20%, of mature parenchymal cells and under 5% for stem/progenitors, and with donor cells distributing also to ectopic sites such as the lungs (Fox and Chowdhury, 2004; Ito et al., 2009). The pioneering efforts of Habibullah and associates found that they could transplant hepatic stem cells safely *via* the hepatic artery (an impossible option for mature parenchymal cells given their size) and were able to achieve 20–25% engraftment efficiency and significant improvement in clinical conditions of their patients but still with ectopic cell delivery as a persistent concern (Aleem Khan et al., 2006; Khan et al., 2010; Parveen et al., 2011). The breakthrough by Habibullah and associates has not been followed by more extensive exploration of such approaches because both of concern of ectopic cell delivery of donor cells and of the need to use fetal liver-derived stem/progenitors to minimize immunological rejection and with difficulties in obtaining and using fetal tissues.

Cell therapies have shown efficacy in preclinical models using transplantation of hepatocytes, mesenchymal stem cells, and macrophages (Forbes et al., 2015). However, few positive results in animal models have been translated through to successful clinical therapies.

Most recently, efforts are being made to adapt grafting methods for transplantation into solid organs (Turner et al., 2010). These include cell sheet engineering technologies that involve attachment of a cell sheet prepared *ex vivo* to the surface of the liver and able to provide some functions to overcome deficits (Kikuchi and Okano, 2005; Tatsumi and Okano, 2017). The more powerful approach has been use of biomaterials supportive of stemness traits of the donor cells (Lozoya et al., 2011) in conjunction with injection grafting (Turner et al., 2013) or patch grafting (Zhang et al., 2021), strategies that enable transplantation of large numbers of cells or of organoids that can correct major disease states. Moreover, they have proven successful for engraftment even in normal liver conditions, thus enabling avoidance of the requirements for a cellular vacuum created by a genetic condition or drug. These new approaches are in their infancy and so must be explored further to assess their real potential.

The treatment of liver damage through liver cell transplantation is key to current research in the field of regenerative medicine and with the potential to dominate treatment in the future, especially if achieved with transplantation using grafting strategies. Transgenic mice provide an ideal research tool for evaluating the functionality of cells after hepatocyte or stem cell transplantation (Table 6). The most utilized systems are those of mice with the absence of the Fah gene (fumaryl-acetoacetate hydrolase) and several genetic derivatives (*Fah* ^−/−^ *Rag2* ^−/−^, *Fah* ^−/−^ *Rag2* ^−/−^ *IL2Rg* ^−/−^), all of them associated with tyrosine degradation.

**TABLE 6:** Mouse models of cell transplantation and liver regeneration.

Mouse model	Method procedures	Pathological changes	Types of injuries	Strengths	Weaknesses	References
HSVtk transgenic mouse	6 mg/kg ganciclovir intraperitoneal injection	HSVtk expressed by hepatocytes promotes ganciclovir phosphorylation and promotes specific ablation of hepatocytes	Acute or chronic liver injury	High repopulation efficiency, NOG background, broad treatment time windows for toxicology evaluation	Low reproduction efficiency	Kosaka et al. (2013)
uPA-transgenic mouse	uPA is structurally expressed and aggregated in hepatocytes under the control of an albumin promoter	uPA is continuously expressed under the drive of Alb, causing apoptosis and persistent liver damage	Acute liver injury, liver failure	Functional human hepatocyte and high efficiency of liver engraftment	High morbidity in neonatal mouse and clearance of uPA gene	Heckel et al. (1990)
*Fah* ^*−/−*^ mouse	Fah-negative, can be maintained by NTBC administration	Toxic metabolites of tyrosine accumulate in the liver and kidneys to induce persistent damage of hepatocytes	Acute and chronic liver injury (depending on time and concentration of NTBC)	Selective advantages during transplantation, controllable liver injury	The NTBC administration process may interfere with liver regeneration or drug metabolism	Overturf et al. (1996)
*Fah* ^*−/−*^ *Rag2* ^*−/−*^mouse	Breeding *Fah* ^*−/−*^ mice with *Rag2* ^*−/−*^ mice; maintained by NTBC administration	Toxic metabolites of tyrosine accumulate in the liver; depletion of B,T cells; depletion of NK by anti-asialo GM1		Ideal for reproduction, sufficient number of homozygotic mice; High repopulation efficiency		He et al. (2010)

### Alb-uPA Mouse Model

The first model used for liver repopulation by donor cells was the albumin-urokinase type plasminogen activator (Alb-uPA) mouse model established in the Ralph Brinster lab (Sandgren et al., 1991). The mouse was originally developed in 1990 as a model for studying bleeding disorders in newborns (Heckel et al., 1990). In Alb-uPA mice, uPA is structurally expressed in hepatocytes under the control of an albumin promoter, leading to intracellular lysis of plasminogen into active plasmin, causing proteolysis within hepatocytes, promoting activation of apoptosis, and ultimately leading to persistent liver damage and failure. Mercer DF and his colleagues hybridized Alb-uPA and CB-17/SCID/bg mouse strains and constructed approximately 70% humanized uPA/SCID mice with spleen transplantation. Transplanted human liver cells expanded about 1,000-fold (Rhim et al., 1994; Mercer et al., 2001). However, uPA/SCID mice have problems such as decreased human hepatocytes implantation rate due to deletion of the uPA transgene by homologous recombination, limited numbers of mice available, small mouse size, and kidney damage phenotype and high mortality. To address these drawbacks, Tateno et al. (2015) developed the so-called cDNA-uPA-SCID model, a mouse model expressing the uPA gene cDNA that allows human liver cells to reproduce. At the same time, more studies began using *Fah* ^*−/−*^ mice, a mouse model that mimics type I Tyrosinemia metabolism disorders, as the primary mouse model for liver cell transplantation studies.

### *Fah* ^*−/−*^ Mouse Model

Fumarate acetate hydrolase gene knockout (*Fah* ^*−/−*^) mice have lost the production of fumaryl-acetoacetate hydrolase, resulting in the accumulation of succinyl acetone in the liver and causing liver damage. *Fah*-deficient mice can maintain normal survival by daily administration of NTBC drugs. Removal of NTBC usually results in the animals dying within 2–3 weeks if very young mice (e.g., 4-week-old hosts) and dying after ∼4–5 weeks in hosts that are older (>3–4 months of age). The model was originally used in pathophysiology studies (Overturf et al., 1996). Later researchers found that Fah-positive cells have a strong selective growth advantage in the *Fah* ^*−/−*^ liver. Similar to the results in the uPA transgenic mouse model, transplanting healthy mouse liver cells, mice can result in rescue from toxic damage to loss of fumarylacetoacetate. After transplantation of parenchymal cells, when under the conditions of animals treated with NTBC, wild-type parenchymal cells form scattered clusters of FAH-positive cells in the FAH-negative liver. However, once NTBC is withdrawn, donor cells can proliferate extensively, form large FAH-positive clusters within 3 weeks and replace almost the entire liver parenchyma of Fah^−/−^ mice within 6 weeks post-transplantation. In parallel, *Fah* ^*−/−*^ mice remain healthy per their liver functions and histological architecture of organs such as liver and kidney. In addition, since the exon five in the *Fah* gene has been completely deleted, no genotype changes will happen during homologous recombination. Therefore, compared with other models, this mouse model has excellent reproductive advantages, which is easy to maintain with relatively low costs.

### *Fah* ^*−/−*^ *Rag2* ^*−/−*^ *IL2Rg* ^*−/−*^ Mice

To expand the application of *Fah* ^*−/−*^ mouse models for the assessment of human hepatocytes, Grompe et al. developed the *FRG* triple knockout (*Fah* ^*−/−*^ *Rag2* ^*−/−*^ *IL2Rg* ^*−/−*^) mice by hybridizing *Fah* ^*−/−*^ mice with *Rag2* and *IL2rg* immunodeficiency mice (Azuma et al., 2007). Deletions in *Rag2* and *IL2-Rg* in mouse prevent the development of B and T cells as well as NK cells. Thus, *Rag2* ^*−/−*^ *Il2rg* ^*−/−*^mice have been previously shown to be excellent hosts for xenografts of human hematopoietic cells as well as for hepatocytes. Due to deficiencies in the immune system, *FRG* mice allow for efficient reproduction of human liver cells (Traggiai et al., 2004). Currently, *FRG* mice are also widely used to detect the reproduction capacity of cultured mpPHHs (mouse-passaged primary human hepatocytes) and hepatocyte-like cells (HLCs) derived from various sources (Michailidis et al., 2020). The repopulation rate of human hepatocytes in *FRG* mice is ∼30%. By using retrorsine, a member of the pyrrolizidine alkaloid (PA) family of naturally occurring compounds that are toxic to various mammalian tissues, Michailidis et al. improved the humanization of chimeric mouse livers, which increased the reproduction efficiency of PHH in *FRG* mouse livers by an average of 10 times. In theory, PHH isolated from humanized *FRG* mice can be used for subsequent *in vitro* cultures or for transplantation, which greatly expands the donor cells when PHH is limited. However, comparing with *Fah* ^−/−^ mice, the liver damage of *FRG* mice causes higher death rate therefore requires higher housing environment criteria, and are more difficult to manage.

### *Fah* ^*−/−*^ *Rag2* ^*−/−*^ Mice

**FIGURE 2:**
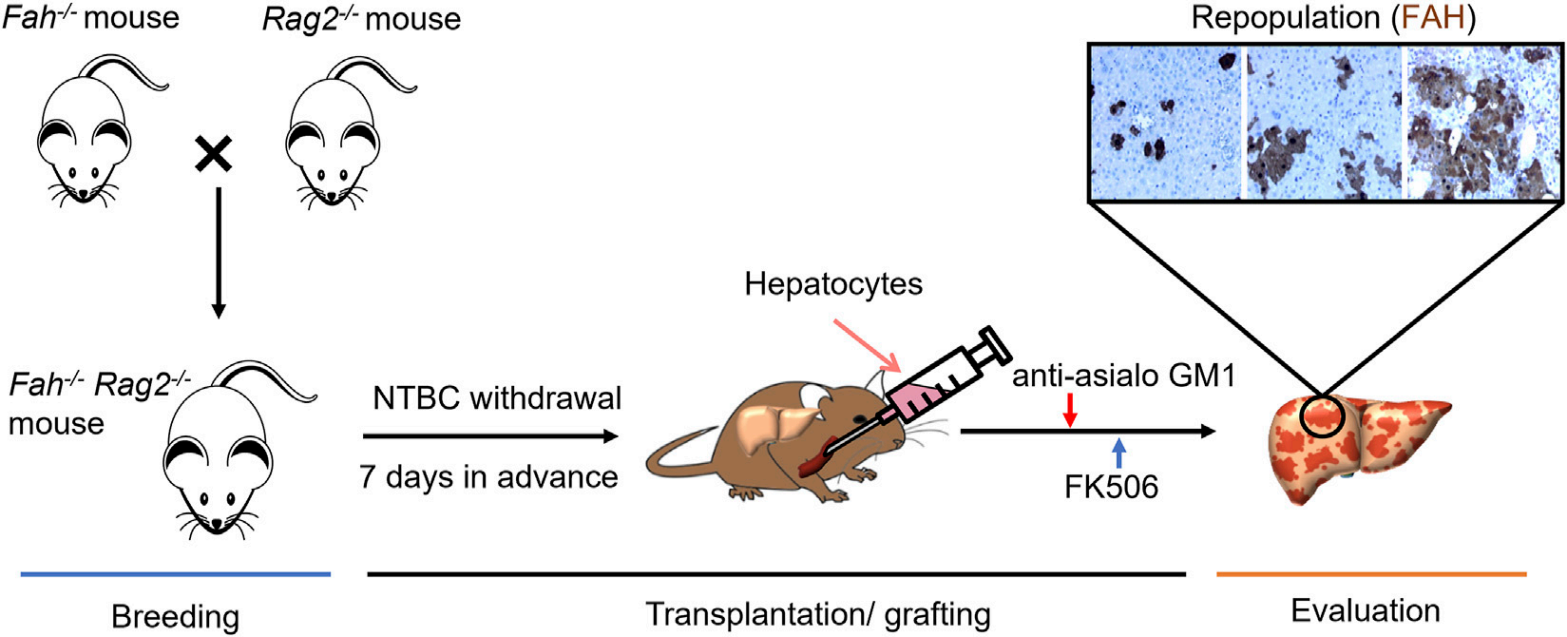
Development of *Fah* ^*−/−*^ *Rag2* ^*−/−*^ Mouse Model and its Application for Functional Evaluation of Hepatocytes. Breeding: *Fah* ^*−/−*^ *Rag2* ^*−/−*^ mouse were generated from the cross breeding of homozygous *Fah* ^*−/−*^ mouse and *Rag2* ^*−/−*^mouse. The breed is maintained as *Fah* ^*−/−*^ *Rag2* ^*−/−*^ or *Fah* ^*−/−*^ *Rag2* ^*+/−*^ with daily NTBC water supply. Transplantation/grafting: Liver injury condition developed 1 week before transplantation by withdrawing NTBC with a stepwise protocol, primary hepatocytes or generated from other sources can then be transplanted *via* blood fusion or now by patch grafting. Anti-asialo GM1 and FK506 were then gave daily for promoting the engraftment of donor hepatocytes. Evaluation: Repopulation rate of donor hepatocytes in *Fah* ^*−/−*^ *Rag2* ^*−/−*^ liver can be confirmed by IHC staining of FAH.

He et al. identified and established *Fah* ^*−/−*^ *Rag2* ^*−/−*^mouse strains by mating and breeding *Fah* ^*−/−*^ mice with *Rag2* ^*−/−*^ mice (He et al., 2010). Compared to *FRG* mice, *Fah* ^*−/−*^ *Rag2* ^*−/−*^mice are able to mature normally and to be used for reproduction, providing a large number of homozygotic mice to be used. Based on this mouse strain, a hepatocyte transplantation protocol and an *in vivo* functional evaluation system of human hepatocytes were established (Wang et al., 2018). For ensuring a higher transplantation rate of human hepatocytes in FR mice, He et al. combined the protocol of gradually withdrawing NTBC in combination with treating animals with immunosuppressant FK-506 and anti-asialo GM1 antibodies before transplantation. The repopulation rate of human hepatocytes in mice liver is up to 70–90% (Figure 2) (Su et al., 2011). For further evaluation of the functions of transplanted hepatocytes, several liver’s parameters such as the viability, values of body weight, liver function index, expression levels of human hepatocyte-specific proteins, and the capacity of HBV infections were collected and combined to be used as evaluation criteria. In He’s study, the transplantation protocol and the evaluation system were applied to compare the functions of human hepatocytes and human fetal liver cells for their engraftment efficiency and functional rescuing potentials. Results showed that both adult human hepatocytes and fetal liver cells were capable to proliferate *in vivo* after transplantation into *Fah* ^*−/−*^ *Rag2* ^*−/−*^mice, and the proliferating fetal liver cells developed primary hepatocyte functions and could be infected with human HBV virus (Wang et al., 2018). The establishment of *Fah* ^*−/−*^ *Rag2* ^*−/−*^mouse strains and related technical systems expanded the application of *Fah* ^*−/−*^ mouse model and is the current most ideal and generally utilized animal model for evaluating the function of human hepatocytes.

### Other Liver Regeneration Mouse Models

In recent years, new liver damage mouse models have also been developed. The TK-NOG mouse model is one of them, which utilizes albumin promoters to drive the HSVtk gene in severely immunodeficient NOG mice (Kosaka et al., 2013). When given non-toxic doses of ganciclovir (GCV), HSVtk, expressed by hepatocytes, promotes phosphorylation of GCV, which in turn causes hepatocyte-specific ablation. Studies have shown that the reproduction efficiency of human hepatocytes in TK-NOG mice is higher than that of FRG mice, but lower than that of uPA-SCID mice (Hasegawa et al., 2011). However, compared with uPA-SCID mice, TK-NOG mice can provide a more stable humanized model, and the reproduction efficiency of hepatocytes does not gradually decrease (Table 6).

In another study, to induce apoptosis of host hepatocytes and promote implantation and replication of transplanted hepatocytes, the researchers inserted active Caspase 8 fused with the FK506 binding domain (FKBP) after the Alb promoter. In parallel, CD34+ human hematopoietic stem cells (HSCs) were prepared. Co-transplantation of human hepatic stem/progenitor cells (Hep) and CD34+ hemopoitic stem cells (HSCs) into transgenic mice and treatment with FKBP dimer AP20187 resulted in the construction of a novel, humanized double chimeric mouse model (AFC8-hu HSC/Hep) with a human immune system and human hepatocytes for the study of the immune pathogenesis of hepatitis C virus (HCV) (Washburn et al., 2011; Dorner et al., 2011; Bility et al., 2012). Unfortunately, human hepatocyte reproduction efficiency in AFC8 mice is lower than that in uPA-SCID and FRG mice.

## Summary and Prospect

An ideal animal model of liver injury should have the following characteristics:

1) Analogue: Animal models should be able to recapitulate approximately the pathological changes that occur in human liver damaged conditions.
2) Reproducibility: the modeling process should be able to be constructed varying from different individuals, different experimental sites, etc., and the experimenter should be able to obtain a uniform molding effect according to the standard experimental protocol.
3) Reversibility: When given appropriate treatment, the model animal should regain its health or obtain an effective prolongation of survival.
4) Adequate treatment window: When model animals develop liver damage or even liver failure, there should be sufficient treatment time to obtain credible experimental results.
5) Appropriately sized animals: Model animals should be large enough to obtain adequate blood and tissue samples.

At present, mouse models of liver injury have been widely used in liver regeneration and repair, cell transplantation treatment, drug screening and evaluation, and have become a necessary part of the safety and efficacy evaluation of new treatment methods. In this review, we did not explore mouse models of bile duct injury, and there are already some mouse models of bile duct injury achieved by dietary regulation or gene knockout, but these models lack specificity, and most of them have a length of time needed for establishment and have, unfortunately, poor stability. Although the research on liver-damaged mice has made relatively gratifying progress, there are still many problems to be solved.

For example, because liver fibrosis is the common outcome for many chronic liver injury conditions, the current available drug-induced fibrosis models or humanized mouse models can present only part of the clinical scenarios. There are so far no available fibrosis models due to alcohol injuries or chronic HCV infections. Also due to the rapid regeneration rate of the mouse liver, most of the models lose their fibrosis phenotypes and recover to a normal histological status within days. This instability reduces the value of those models for drug screening or treatment development.

Also, although there are already humanized liver mouse models for the study of HCV and HBV infections, due to the differences in the immune systems of mice and humans, the costs of the models is expensive, and their performances need to be further improved. Nevertheless, the establishment of these mouse models to meet the criteria required for human liver viruses will have great application potential. Mouse models have made good progress in liver injury repair and liver regeneration mechanisms, combined with the further application of technologies such as deep sequencing and gene editing, it is believed that more mouse models suitable for different types of liver damage research will gradually be established.
